# Designing an AI-Based Virtual Try-On Web Application

**DOI:** 10.3390/s22103832

**Published:** 2022-05-18

**Authors:** Davide Marelli, Simone Bianco, Gianluigi Ciocca

**Affiliations:** Department of Informatics, Systems and Communication, University of Milano-Bicocca, Viale Sarca 336, 20126 Milano, Italy; simone.bianco@unimib.it (S.B.); gianluigi.ciocca@unimib.it (G.C.)

**Keywords:** virtual try-on, 3D face reconstruction, artificial intelligence, deep learning

## Abstract

In the last few years, Augmented Reality, Virtual Reality, and Artificial Intelligence (AI) have been increasingly employed in different application domains. Among them, the retail market presents the opportunity to allow people to check the appearance of accessories, makeup, hairstyle, hair color, and clothes on themselves, exploiting virtual try-on applications. In this paper, we propose an eyewear virtual try-on experience based on a framework that leverages advanced deep learning-based computer vision techniques. The virtual try-on is performed on a 3D face reconstructed from a single input image. In designing our system, we started by studying the underlying architecture, components, and their interactions. Then, we assessed and compared existing face reconstruction approaches. To this end, we performed an extensive analysis and experiments for evaluating their design, complexity, geometry reconstruction errors, and reconstructed texture quality. The experiments allowed us to select the most suitable approach for our proposed try-on framework. Our system considers actual glasses and face sizes to provide a realistic fit estimation using a markerless approach. The user interacts with the system by using a web application optimized for desktop and mobile devices. Finally, we performed a usability study that showed an above-average score of our eyewear virtual try-on application.

## 1. Introduction

With the development of advanced technologies in the last few years, Augmented Reality (AR) and Virtual Reality (VR) have emerged as key technologies in different interactive application domains. For example, they are utilized in the gaming industry [1], for human–computer interaction [2,3], and in the manufacturing industry [4,5].

Recently, they have been increasingly employed in applications designed for physical and online retail stores [6,7]. In the retail scenario, AR and VR are technologies that a consumer interacts with and directly experiences while in the physical store, or while navigating the virtual online store. These technologies enhance the user experience during the purchase by allowing them to interactively browse, study, or try the products before buying them [8]. This is especially true for online shopping, where the users cannot directly interact with the products they are interested in. According to Statista, “online shopping is one of the most popular online activities worldwide, and e-commerce sales in 2019 amounted to USD 3.53 trillion and are projected to grow to USD 6.54 trillion in 2022” [9]. Similar trends can be found in other countries as well. According to research by Gartner, “46 percent of retailers plan to deploy either AR or VR solutions to meet customer service experience requirements” [10].

Appealing to the users with novel and engaging advanced AR and VR applications is thus a key factor in a market that is growing to such an extent. Examples of such applications are smart mirrors and virtual try-on systems [11,12,13,14]. Smart mirrors are devices designed to interact with users in a different way. They are often based on the Internet of Things (IoT) concept and allow users to interact with applications using touch or voice controls and to display feedback and various information [15].

Although smart mirrors are general-purpose devices with a wide range of applications, here, we are more interested in virtual try-on systems that are instead specifically designed to effectively improve product visualization and pre-purchase information for the retailers, and to enhance the entertainment value of the shopping experience for the end-users.

The design of a virtual try-on system has several challenges—for example, how to present to the user the result of the try-on. Once the user selects the item to wear, it can be shown in real time or in a deferred mode. In the first case, a live-feed video is usually the preferred solution. The user can move and see how the item fits his needs, and interact with the system. In the second case, a processed video is shown to the user, who can browse it. The video can be generated offline, and the user has limited interactivity with the system.

Regardless of the modality chosen, advanced processing software modules to support the user experience are required. For example, it is necessary to recognize the presence of the user (e.g., using face detection or people detection algorithms), the pose has to be taken into account to virtually dress the user (i.e., pose recognition), and occlusions may occur and should be dealt with (i.e., occlusion detection). Finally, the rendering of the virtual item in the real-world scene should be as seamless and plausible as possible.

There are many methods to computationally address the above-mentioned challenges. In recent years, computer vision has taken great leaps thanks to advances in artificial intelligence and machine learning. In particular, deep learning and artificial neural networks have served to boost the development of robust and fast computer vision-based algorithms, which are effectively and efficiently exploited in many application domains [16,17].

In this paper, we describe a framework for the design of an eyeglasses and sunglasses virtual try-on web application that leverages advanced deep learning-based computer vision techniques. Specifically, to allow the users to easily see how the eyeglasses look interactively, the application exploits a 3D face reconstruction algorithm from a single input image. In this way, we can show a 3D virtual face of the user with the selected eyeglasses, and the user can pose it and view the rendered eyeglasses from different viewpoints. This is particularly useful when the user suffers from view problems and thus has difficulties checking the appearance of glasses when not wearing lenses. Actual face and eyeglasses sizes are taken into account to provide a realistic fit feel. To maximize the user experience, the try-on process is fully automated and only requires a face picture and the selection of the glasses model to test. The work presented here is the completion of our previous investigations in the design of virtual try-on systems [18,19]. The framework described here is a generic solution that can be used as a blueprint to build other, extended and specialized, try-on applications.

The paper is organized as follows. In Section 2, we briefly review existing virtual try-on applications and the most recent techniques for 3D face reconstruction, which is at the basis of the try-on application. In Section 3, we describe the workflow of a generic virtual try-on system, highlighting the relevant modules, interactions, and dependencies. We use the generic workflow as a blueprint to design our virtual try-on application for eyeglasses and eyewear. The design process starts by assessing the existing 3D face reconstruction approaches in the literature. In Section 4, we analyze and compare their design, complexity, geometry reconstruction errors, and reconstructed texture quality. Our proposed system is then described in Section 5. In Section 6, we describe how to evaluate the try-on application from the user experience perspective. Finally, Section 7 concludes the paper.

## 2. Related Works

The focus of this paper is the virtual try-on of eyeglasses and sunglasses. Therefore, in the first part of this section, we review the existing virtual try-on solutions for face accessories and makeup, discussing their strengths and weaknesses. Then, since an essential step in our try-on is the 3D face reconstruction, we briefly overview the existing reconstruction methods, particularly those working with a single face image as input.

### 2.1. Virtual Try-On Applications

In the last few years, there has been a large increase in demand for the development of virtual try-on applications by commercial companies. The development of such applications has been made possible by the advances in AR and VR technologies. The first time that AR technology reached the broader public was in 2016, when Niantic launched Pokémon GO, an AR-enhanced game available both to iOS and Android users. Later, the use of AR was democratized even more, after popular smartphone apps such as Snapchat, Instagram, and Facebook developed their new AR filters and, more recently, after Google launched AR objects on Google search and introduced a set of functions for face detection, 3D reconstruction, and augmented reality in the ARCore SDK [20].

The need for virtual try-on applications has further increased in the last couple of years due to the pandemic, which made it impossible for customers to participate in a physical try-on in stores.

These virtual try-on solutions are usually available as mobile or web applications, and have the objective to allow a potential customer to virtually try on himself some products sold by a store or manufacturer, giving him an experience similar to the one he would have in a physical store. In the following, we briefly review the arguably most popular virtual try-on solutions for face accessories and makeup:

- Ditto’s virtual try-on [21]—this is a 3D eyeglasses and sunglasses try-on application that pays particular attention to the actual sizes. It uses a library of glasses and fits them to the estimated user’s face size. The application can also recommend the best-looking glasses for the user. For the try-on process, the user is asked to follow specific instructions. He has to record a short video while rotating the face horizontally. A credit card-sized object placed on his forehead is exploited to estimate the face size. The try-on result is shown by rendering the glasses on multiple video frames with a different face orientation.
- XL Tech Apps’s Glassify [22]—this is a virtual try-on application that works with a single frontal face image. The application requires the user’s intervention in several steps: first, the user chooses the shape that best fits his face; then, the software fits the eyeglasses on his face; finally, the user manually adjusts the position and scale of the glasses over the picture. This application works correctly only with frontal face images, and only the forepart of different glasses models can be rendered over the input image.
- Perfect Corp’s YouCam Makeup [23]—Perfect Corp’s YouCam Makeup is a virtual try-on application mainly conceived for makeup but can also be used to virtually change hair color, hairstyle, and accessories (e.g., jewelry and glasses). The framework also includes a virtual beauty advisor, as well as tools for face detection, face tracking, and augmented reality. Some of the try-on features work in real time on the live camera video stream. The application presents some limitations in the glasses try-on: it renders only the front frame without the temples and frequently fails to properly fit the glasses when the face is not in a perfect frontal position.
- MemoMi’s Memory Mirror [24]—this is an application that works differently from the previous ones. It is mainly conceived to be used in physical stores, and it requires the user to wear real eyeglasses in front of a magic mirror used to record a video of the user while trying different accessories or makeup. Each tested element can be reviewed by replaying the video, and comparison with other products is possible by displaying two videos side by side. The main limitation of this solution is that the need for specific hardware and real glasses makes it not suitable for try-on outside of stores.
- Jeeliz [25]—this is an application for real-time web-based glasses virtual try-on. The application is available as a JavaScript widget, thus permitting integration with glasses virtual try-on in a website or a mobile web application. The application renders the 3D model of the glasses in real time on the live camera video stream. The user sees his face as in a mirror, but with glasses. There are some limitations: the tracking of the face is slow, and the glasses positioning has some delays; it uses only the front frame and the very first part of the temples, which very often penetrate the user’s face.
- Luxottica’s Virtual Mirror [26]—this solution provides eyewear try-on in real time on a camera video feed. The user has to stand still, in a frontal position, looking downwards, for face detection and the 3D glasses model positioning on the face. The rendering follows the movements of the head and gives a digital reflection on the lens and frame for increased realism. The main limitation is that the fit of the glasses to the user’s face is not automatic and can only be manually adjusted using a dedicated button.
- Safilo VirtualEyes [27]—this is an application that can photo-realistically render, in Augmented Reality, a vast selection of glasses on any surface. The application exploits Safilo’s 3D eyeglasses models, which have been optimized through the analysis of the ambient light in order to achieve a realistic effect. The main limitation of this solution is that the glasses can be only rendered on a surface and not on the user’s face.

The vast majority of the above solutions are available as standalone applications and frameworks integrable with existing services and platforms. Most of these applications offer integration with social services to allow the user to share their virtual try-on sessions and with store platforms to allow the user to buy the products. The key features of the virtual try-on systems reviewed above are summarized in Table 1 for ease of comparison.

### 2.2. 3D Face Reconstruction

Three-dimensional face reconstruction, as well as 3D reconstruction in general, is a long-standing problem, usually requiring specific hardware [28] or multiple images [29,30,31]. Since we want to provide the user with an easy-to-use application for virtual try-on, we aim to simplify the face acquisition and 3D face reconstruction processes as much as possible. Therefore, we focus on 3D face reconstruction methods that only need a single image as input, with particular emphasis on AI-based approaches, which are able to learn from data both the best reconstruction parameters and more robust prior information. The most common existing methods for 3D face reconstruction from a single image can be grouped into two main categories: *3D Morphable Model (3DMM) fitting-based* and *shape regression-based*.

The use of 3D Morphable Models (3DMM), introduced by Blanz et al. [32], represents a common solution to the 3D face reconstruction problem from a single view. Within this category, Huber et al. [33] searched for the correspondence of local features on the images and landmarks on the 3DMM. These correspondences are then used to regress the 3DMM deformation coefficients that generate a 3D face mesh similar to the one in the image. More recent methods belonging to this category, such as that of Tuan et al. [34], use Convolutional Neural Networks (CNNs) to learn to regress the 3DMM coefficients.

The main advantage of the solutions exploiting 3DMM is the availability of the complete face 3D model under any circumstance, since even the parts that are occluded in the input image are reconstructed using the 3DMM’s geometry. On the other hand, the main limitation of these 3DMM-based solutions is that the reconstruction is often too similar to the original 3DMM model. This means that the characteristic facial traits normally defined by small details of the facial geometry are not usually well reconstructed.

A recent approach based on 3DMM models, proposed by Ranjan et al. [35], claims to be able to obtain better results than previous works, especially for the reconstruction of facial expressions. To this end, they exploit the FLAME [36] 3D face base model representation. To cope with the lack of details in the reconstructed face, Extreme3D [37] introduced a bump map to model wrinkles on the Basel Face Model (BFM) [38]. The bump map applies geometric deformations on the reconstructed 3D face, and a symmetry constraint is used to cope with occlusions. The Ganfit method proposed by Gecer et al. [39], and its fast extension [40], use a different approach and generate a realistic face shape and texture by means of Generative Adversarial Networks (GANs). The method can reconstruct very accurate models but is not available for general use. In order to better fit the generic 3DMM model to the actual face, researchers have tried to integrate different properties and constraints into their training pipelines. For example, using deep networks, Deep3DFace [41] regresses several coefficients that are used to model and fit the face: the identity of the user, expression, texture, pose, and lighting. Coupled with photometric and perceptual losses, they are able to better fit the actual user’s face. RingNet [42] is also a deep network that regresses model parameters. Specifically, it regresses the shape, pose, and expression of the FLAME model. Differently from the previous methods, RingNet exploits multiple images of the same person and an image of a different person during training to enforce shape consistency. Zhu et al. [43] proposed a Fine-Grained Reconstruction Network (FGNet) that can concentrate on shape modification by warping the network input and output to the UV space, achieving a final reconstruction that includes fine-grained geometry. Lin et al. [44] proposed to refine the initial texture generated by a 3DMM-based method with facial details from the input image. To this end, they use graph convolutional networks to reconstruct the detailed colors for the mesh vertices. The space of the parameters that need to be regressed for a 3DMM model is high-dimensional, i.e., 230 values. Guo et al. [45] propose instead to use a small subset to speed up the fitting process. Moreover, a meta-joint optimization is introduced during the training phase to improve the overall fitting accuracy. The final method is able to perform face reconstruction faster than in real time using a MobileNet as a light backbone. The reconstructed 3D face can be used in VR applications—for example, to animate virtual avatars. However, most of the approaches are not able to reconstruct faces that can be animated in an accurate way. DECA [46] has been specifically designed to regress accurate 3D face shape and animatable details that are specific to an individual and that can change according to the subject’s expressions. The method is based on the FLAME face model. Another approach that has been designed with animation in mind is INORig [47]. The approach is built on an end-to-end trainable network that first parameterizes the face rig as a compact latent code with a neural decoder, and then estimates the latent code as well as per-image parameters via a learnable optimization.

The methods in the second category, i.e., shape regression-based methods, were developed to obtain a more accurate reconstruction than the 3DMM-based ones. Among the methods in this category, Jackson et al. [48] propose to straightforwardly map the input image pixels to a full 3D face structure in a voxel space via volumetric CNN regression. The main advantage of this approach is that the output face shape is not restricted to a face model space, therefore permitting applicability to the reconstruction of other objects, e.g., [49]. A similar idea is exploited by Feng et al. [50], who directly regress the face mesh from the input image without making use of a voxel space: the advantage of this method, called PRNet, is that it is lightweight and fast to execute, and also it usually leads to a precise and detailed reconstruction. Guo et al. [51] proposed the use of different CNNs to reconstruct the coarse-scale geometry and the fine detail. Recently, Wang et al. [52] proposed a framework that solves the problem of face reconstruction in three steps: face region segmentation, coarse-scale reconstruction, and detail recovery. Finally, Wang et al. [53] propose a novel unsupervised 3D face reconstruction architecture by leveraging the multi-view geometry constraints to train accurate face pose and depth maps. Once trained, the approach is able to perform reconstruction from a single image as well.

## 3. Workflow of a Generic Virtual Try-On System

**Figure 1:**
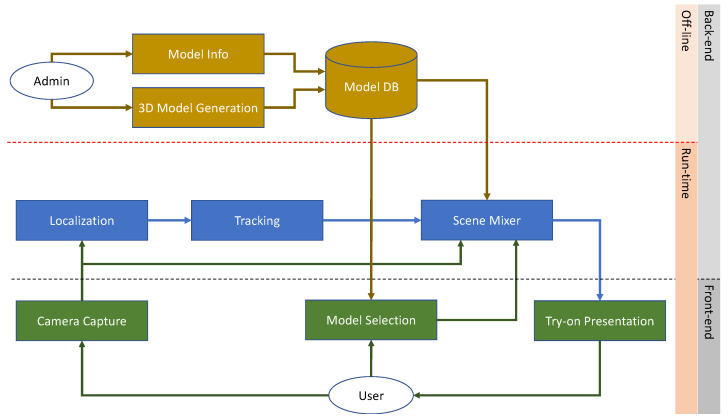
Workflow of a generic virtual try-on system.

Figure 1 illustrates the workflow of a generic virtual try-on system. It shows the essential components and modules of the system and their interactions. The described components and modules are intended to be generic. Different applications can require specific modules or sub-modules to operate.

A generic virtual try-on system can be composed of a set of front-end modules responsible for the management of the user interaction with the system, and a set of back-end modules that implement the system logic and operational activities. Moreover, the modules and activities can be further categorized with respect to their usage. We have offline activities usually performed either at the system’s initialization or periodically to update the system. We also have real-time activities performed while the system is running and the user interacts with it. We will now briefly describe the role of the components and modules depicted in Figure 1.

In the back-end, offline group of modules, we can find all the administrative activities usually involved in the creation of all the data required for the virtualization of the items to be displayed to the user. These activities comprise the collection of the metadata of the items (i.e., attributes, prices, descriptions, etc.) and the generation of the corresponding virtual models. These models can be of different types, but a 3D model is usually required if the items are shown in different poses. The 3D models can be directly generated from CAD files if they are available, can be created from scratch by a graphical designer, or can be acquired using 3D reconstruction techniques such as structure from motion-like approaches [31]. These activities are periodically performed to add and/or remove items from the collection. All the generated data and information are stored in the system (e.g., in a database) and made available to the other modules that operate at run-time.

Concerning the front-end modules that operate at run-time, we have a camera capture module that usually continuously acquires pictures from the environment and streams them to the back-end modules to be processed. Depending on the back-end processing, the camera can capture RGB images (i.e., visible light) or RGBD images (visible light + depth information). The latter requires additional specialized hardware to extract depth information from the scene. Usually, RGB images are sufficient for the majority of the applications as modern computer vision techniques coupled with advanced machine learning methods can extrapolate additional information from them.

The front-end modules are also responsible for the management of the user experience with the system. To this end, the system has modules that encapsulate the user interface logic. For example, the user can interact with the system with a touch or touch-less device to choose the items he is interested in, browse the catalog, and display the information associated with the shown items. Once the user has made his choice, the system can display it worn by the user in the virtual scene. The user can interact with it differently depending on the technology used for rendering. For example, the rendered scene can be static (e.g., a single photo), dynamic (e.g., a video stream), or interactive (e.g., a 3D scene). Each of these presentation modalities requires specific modules to operate.

Between the offline, back-end modules and the run-time, front-end modules, we have the operational core of the system composed of the run-time back-end modules. These modules are usually organized in a pipeline. We have indicated three main modules, but in actual applications, some modules can be merged or separated into further sub-modules. Regardless of this, a generic virtual try-on application needs to identify the presence of the user to start the process of creating the virtual scene (i.e., user localization). This can be accomplished using advanced computer vision techniques. For example, if we are interested in the face of the user (e.g., for virtual makeup or eyeglasses try-on), robust face detection and recognition approaches are present in the literature [54,55,56,57,58], and some of them are readily available in open-source software libraries, either exploiting standard feature-based methods or deep Learning-based ones. Examples of these libraries are OpenCV [59] and DLib [60]. If, instead, we are interested in the full body (e.g., for clothing try-on), human body detection can be accomplished using techniques borrowed from the human action and pose recognition research fields, as described in [61,62,63]. In both cases, the user’s privacy should be assured and long-term storage of data must be avoided. This is especially relevant for applications that are installed in real stores. Once the user is detected, the tracking module is activated. The role of the tracking module is to follow the user’s movements (i.e., the pose) in time. This is necessary to ensure temporal coherence in the display of the virtual scene. In fact, while the user moves in the real world, the virtual item should be positioned and superimposed onto the user coherently in the virtual scene. The tracking module can provide information about the user’s pose in the form of facial key-points [64] in the case of the face, or skeleton points [65] in the case of the body. This information is then passed to the scene mixer module for the generation of the virtual scene.

The scene mixer module collects information from several other modules and uses it to create the final virtual scene rendered and displayed to the user. In order to generate the virtual scene, it is necessary to blend the real one acquired by the camera, the information about the user’s pose, and the item chosen by the user, which must be transformed according to the user pose. The transformed item’s 3D model is superimposed onto the real scene with the user to create the virtual scene. The composite output of the scene mixer module can either be a static image, a recorded video sequence, or a dynamic virtual scene. In the latter case, the system can provide a live video stream augmented with the virtual item superimposed onto the user in real time. To this end, all the back-end processing needs to be executed as quickly as possible to cope with the user’s movements. Alternatively, the system can generate a completely virtual scene with an avatar of the user wearing the chosen item, allowing the user to inspect the scene freely and see himself from different points of view without restrictions. This approach lessens the requirement for the fast-paced and real-time processing of the back-end modules.

Other challenges in the design of a robust scene mixer module are related to the realness of the virtual scene; for example, the virtual item should be rendered at the correct size depending on the user’s pose. Occlusions may occur and must be dealt with. Moreover, the real and virtual parts of the scene must blend seamlessly as much as possible. Finally, the user must not be constrained in the interaction with the system.

## 4. Comparing the 3D Face Reconstruction Approaches

The core of a virtual try-on application for glasses is undoubtedly the 3D face reconstruction module. In the literature, several approaches differ in their underlying architecture, complexity, and performance. In this section, we compare different 3D face reconstruction approaches that can be potentially used in our virtual try-on application. This comparison can be used by developers and practitioners to select the most suitable approach for their specific task, depending on the operational and environmental constraints of the final application.

Among the possible approaches, we considered those that have made their code publicly available: DECA [46], 3DDFAV2 [45], 3DSFMFace [53], Extreme3D [37], RingNet [42], Deep3DFace [41], INORig [47], and PRNet [50].

We compare the 3D face reconstruction approaches with respect to different criteria: (1) underlying architecture and characteristics; (2) computational costs (both in time and memory consumption); (3) quantitative evaluation of the reconstruction error against a ground truth; (4) qualitative evaluation of the goodness of the reconstructed face texture. These criteria capture different aspects of the reconstruction phase. To the best of our knowledge, no standard quantitative evaluation exists to assess the texture, so we opted for a subjective assessment with a panel of users. Both the geometry and the texture are important for an assessment of the fidelity of the reconstruction and thus the user’s acceptance of the try-on application.

### 4.1. Comparison of the Architectures

Table 2 summarizes the characteristics of the 3D face reconstruction methods. All the methods rely upon Neural Networks to perform feature extraction or parameter regression. The most common backbone used is the Residual Network, which has been successfully exploited in different application domains and is one of the most efficient and robust networks. All the methods need a face detector to locate the facial region and extract facial landmarks to be used for pose estimation and normalization. Any face detector could be used, but most methods include one. Three of them do not include a face detector but assume that the input images have either a set of facial landmarks associated (Deep3DFace), or that the face has been already located and cropped (RingNet) or segmented (3DSFMFace). For these methods, we provide images in the intended format. All the methods, except Extreme3D, provide both geometry and texture as output. INORig is the only one that requires multiple images of the subject as input. All the methods are implemented in Python and are based either on TensorFlow or PyTorch frameworks.

### 4.2. Computational Costs

For each method, we computed the time and memory required to perform the face reconstruction, as well as the geometry error. Table 3 summarizes the results. The execution times reported in the papers describing and comparing the different methods are usually relative only to the 3D face reconstruction step and do not include the preprocessing and postprocessing steps. When building an application such as the virtual try-on, it is more important to consider all the steps involved in the reconstruction process to evaluate the overall run-time. We modified the source codes of the 3D face reconstruction methods to perform a consistent evaluation across them. Each pipeline was initialized once and then used to process iteratively 101 loosely cropped face images from the FFHQ dataset [69]. The execution time for each sample includes all the steps from image loading to the creation of the 3D model representation (i.e., obj or ply files). Since the frameworks use some caching and loading of data on the first run, we decided to discard the execution time of the first image to simulate a hot start of the system. The times of Table 3 are therefore relative to the execution of 100 face reconstructions on a machine with an Intel Core i7 7700 CPU and an NVIDIA Quadro RTX 6000 GPU. Some changes to the source codes were needed due to the differences in the input data and setup required by the methods. For the Extreme3D, we disabled the cuDNN back-end due to incompatibilities with the GPU. Deep3DFace needs five face landmarks as input in addition to the image; we added a preprocessing step to automatically compute such points using the DLib library as suggested by the authors. Moreover, 3DDFAV2 reconstructs all the faces detected in the input picture by design; we forced it to work only on the first detection. The authors of 3DDFAV2 stated that, using the ONNX Runtime [70] library, it is possible to obtain a noticeable increase in the inference speed. Since this optimization library is potentially usable by the other implementations, we decided to use the plain (non-onnxRuntime) version of their method for a fair comparison. INORig requires at least two images of the same person; we modified the code to use the input image and its horizontal flip, as done by the authors.

The majority of the methods can provide face reconstruction in less than one second on average. The only exceptions are INORig and Extreme3D; the former works on two images, while the latter performs part of the computation on the CPU, slowing down the process. The fastest method is 3DSFMFace, although its output is a point cloud and not a 3D mesh, as with the other methods. It also requires a segmented face over the input image, and the reported time does not include the time necessary to perform such segmentation since we achieve this task manually.

We also evaluated system RAM usage and GPU dedicated RAM usage. Table 3 reports the peak memory allocated for both CPU and GPU by each method during the reconstruction of a single 3D face. System memory allocation varies between 2GB and 4GB, depending on the pipeline. On the GPU memory side, the amount of memory used varies between 1.1GB for PRNet and 4.6GB for 3DDFAV2. While most of the implementations have a constant GPU memory usage, DECA and INORig present some short spikes of allocation that bring the peak memory usage to 18GB and 21GB, respectively. The implementation of RingNet seems to allocate all the available GPU memory, even if this behavior is explicitly disabled in the TensorFlow library.

### 4.3. Reconstruction Errors

We evaluated the reconstructed 3D geometries on the UMB-DB dataset [71] to assess the geometrical error of different face reconstruction methods on the same input data. This dataset contains RGB images and the corresponding 3D ground truth geometry acquired using a Minolta Vivid VI-900 laser depth scanner. Reconstructions of 15 subjects were performed for each method starting from a single neutral expression input image without occlusions. Since the methods use different coordinate reference systems, we performed a first coarse alignment to match the reconstruction to the ground truth geometry using the seven face landmarks annotated in the UMD-DB. This rigid alignment only allows the rotation, translation, and scale of the reconstructed geometry. Considering that the completeness of the reconstructed geometry varies between the methods, we decided to crop all the reconstructions to the same area of the face. Given that INORig is the method whose reconstruction includes the smallest portion of the face, we decided to crop out the parts that are not reconstructed by INORig—for instance, the ears (reconstructed by Extreme3D, PRNet, DECA, and RingNet) and the cranium (provided by DECA and RingNet). Another rigid alignment step was performed through the Iterative Closest Point (ICP) algorithm to register the cropped 3D reconstruction to the ground truth. Finally, the geometry was evaluated using the absolute distance between each vertex of the 3D reconstruction and its closest point on the ground truth mesh.

**Figure 2:**
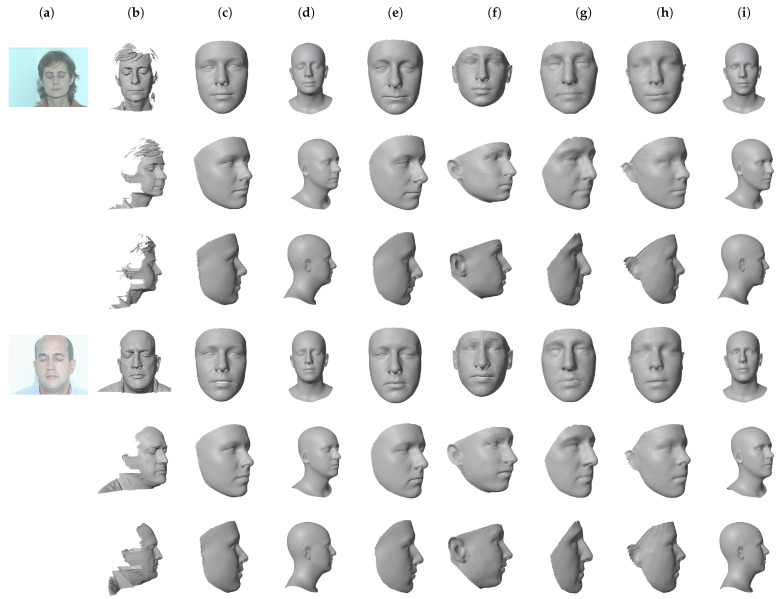
Examples of 3D face reconstruction results on images from the UMB-DB dataset [71]. For DECA, the figure shows the coarse mesh that, opposed to the detailed one, provides a texture. (**a**) Input image; (**b**) ground truth; (**c**) 3DDFAv2 [45]; (**d**) DECA [46]; (**e**) Deep3DFace [41]; (**f**) Extreme3D [37]; (**g**) INORig [47]; (**h**) PRNet [50]; (**i**) RingNet [42].

Table 4 reports the results of the evaluation. Since DECA does not provide a texture for the detailed 3D model, we decided to evaluate the coarse one, which includes the texture image. We did not evaluate 3DSFMFace due to the limited usefulness of the point cloud recovered for a virtual try-on application. As can be seen, all the values are similar and well within a reasonable tolerance for a try-on application. For completeness, we also report in the same table the reconstruction performance of the methods on the NoW dataset as per the NoW challenge benchmark site [72]. See [42] for further details. Extreme3D and 3DSFMFace have not been evaluated on the benchmark. Some examples of 3D face reconstructions are visible in Figure 2.

### 4.4. Texture Quality

As stated before, we need both the geometry and the appearance (i.e., the face texture) to be reasonably accurate for our application. To this end, we selected from the FFHQ dataset [69] a subset of 10 face images as a test set. The images were processed with each method using the code, model, and parameters provided by their respective authors. The output of each method was then evaluated in a comparative way. Given an input 2D image, the different 3D outputs were assessed by a panel of subjects that compared the results against the original image and selected the best 3D output with respect to the fidelity of the reconstructed texture and potential appeal within a try-on application.

**Figure 3:**
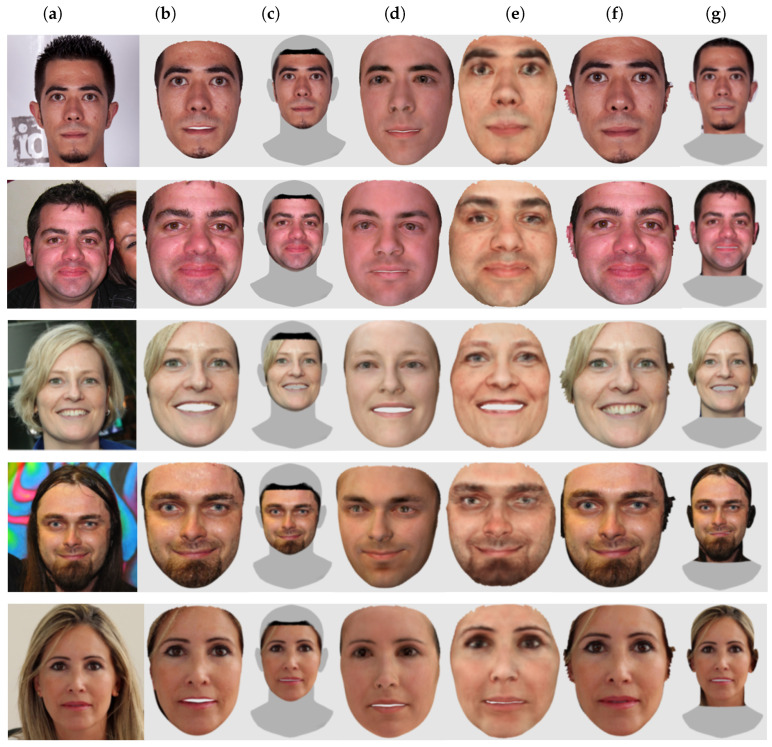
Some samples used in the texture quality experiment. (**a**) Input image; (**b**) 3DDFAv2 [45]; (**c**) DECA [46]; (**d**) Deep3DFace [41]; (**e**) INORig [47]; (**f**) PRNet [50]; (**g**) RingNet [42].

From Table 2, we see that Extreme3D does not output a texture, so it was excluded in the subjective evaluation. Moreover, we excluded 3DSFMFace because it outputs a 3D point cloud and not a mesh. In the end, we compared six methods. For the assessment, we developed a simple web application to show the 3D models reconstructed by the six methods with their texture applied. The users were chosen among researchers and postgraduate students of the University of Milano-Bicocca. All the users had normal or corrected-to-normal vision and were knowledgeable on virtual try-on applications and 3D modeling. The users were asked to rank the results from one (the best) to six (the worst). Ties were allowed. We then collected all the responses and averaged the rankings for each method. In total, 11 users participated in the experiment. Figure 3 shows some of the texture results judged in the subjective experiment. In the web application, the users were able to scale and rotate the models to inspect them more thoroughly.

**Figure 4:**
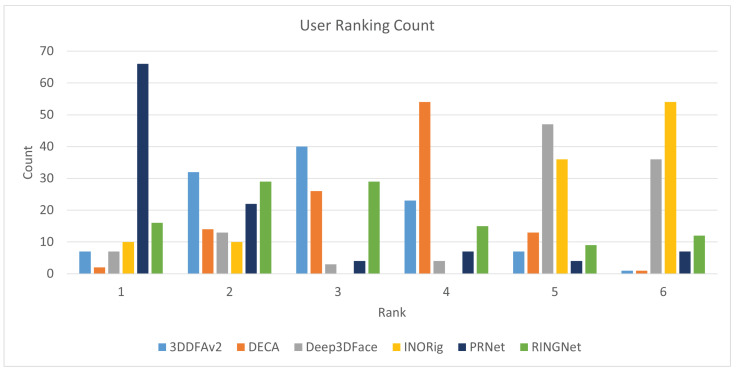
User ranking of the 3D face reconstruction methods in the texture quality experiment.

The average rank of each method is as follows: PRNet: 1.93, 3DDFAv2: 2.95, RingNet: 3.07, DECA: 3.59, Deep3DFace: 4.63, and INORig: 4.85. Figure 4 shows how many times a method was ranked at a given position. Overall, PRNet was judged to provide the best texture on the samples. RingNet and 3DDFAv2 have similar ranks. Next, we have DECA, which has been voted mostly in the fourth position. Finally, Deep3DFace and INORig gave similarly poor results. It was surprising that PRNet was judged the best against more recent methods such as DECA or INORig. This can be explained in that the reconstruction methods are mostly designed with geometry reconstruction as the main goal. The visual texture is usually not considered as the main focus and is used only for visualization. From the experiment, it emerged that one of the problems of existing methods based on 3DMMs is that they tend to create gaps in the mouth when the person is smiling. Since there is no texture for the inner mouth, this creates an uncomfortable hole in the texture. Postprocessing is required to cope with this issue. PRNet, being based on a shape regression technique, has no such problem: the mesh is closed, and the texture is complete. This can be seen in the third row of Figure 3.

## 5. Workflow of Our Virtual Try-On Application

The currently available virtual try-on applications for face accessories present some limitations. For example, many applications show the results of the virtual scene in a static 2D image; this greatly limits the interactivity. Other applications require some kind of physical marker to be acquired along with the user’s face for size estimation. This can be cumbersome in the case of mobile applications. Moreover, using a video stream as output limits the degrees of freedom with which the user can see himself with the accessories.

**Figure 5:**
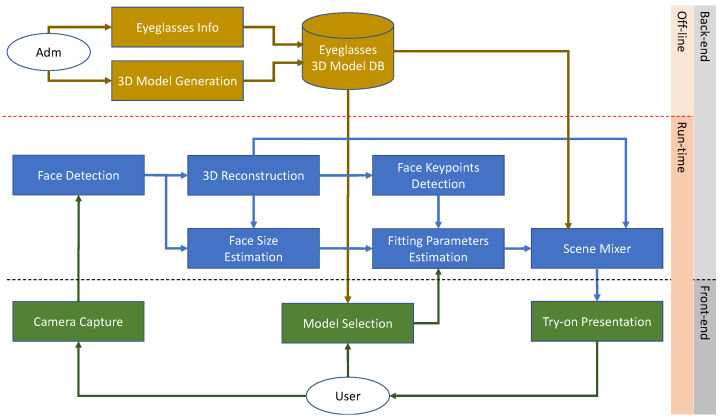
Workflow of our 3D eyeglasses virtual try-on system.

In this section, we will describe our virtual try-on solution designed for eyewear. To overcome limitations in existing try-on applications, our solution leverages artificial intelligence methods, specifically CNNs, for the robust detection and tracking of the face of the user. The solution is designed to be more user-friendly, interactive, and easy-to-use than existing applications. To achieve this, the application creates a complete 3D virtual scene, with the 3D-reconstructed head and face of the user wearing the selected eyeglasses. The reconstruction is performed using a single 2D image of the user’s face. After the 3D reconstruction, the face size and the fitting parameters for the eyeglasses model are automatically computed, leveraging information from the user’s face and without any external physical marker. The result is displayed using a 3D rendering framework that also allows the user to rotate the reconstruction and test on it different glasses models. By using the full 3D results, the user has a realistic idea of how the eyeglasses will look on himself and can freely pose the virtual head, viewing the glasses model from any point of view. Figure 5 shows an overview of the workflow of our try-on solution. The back-end of the system is implemented in the cloud, while its front-end is implemented as a web-based application. In our workflow, the localization module is split into the face detection and 3D reconstruction sub-modules. There is no tracking module since we reconstruct a 3D representation of the user’s face. The face size estimation, face key-point detection, and fitting parameter estimation are supporting modules used to blend the chosen eyeglasses and the user’s reconstructed face with the correct sizes and proportions for a realistic and faithful rendering.

The following subsections provide further details on each component.

### 5.1. Face Detection and 3D Reconstruction from a Single Image

In Section 2.2, we have surveyed several approaches in the state-of-the-art for 3D face reconstruction from a single image. In theory, any of the mentioned approaches can be exploited to build a virtual try-on application. However, we must take into account that in a real application scenario, the approach used must be computationally efficient, and it should reconstruct not only a reasonable 3D geometry of the face, but also an accurate replica of the facial details via a texture to be applied on the 3D model. Without all these constraints, the try-on application would not be appealing to the final users, both in terms of usability and fidelity.

#### Implementation of the 3D Face Reconstruction

The creation of the virtual scene starts with the user’s face detection in a captured photo. To reduce the computational burden, the detection must be executed with a fast and lightweight algorithm. Among the possible algorithms in the state-of-the-art, we selected the CNN-based face detector in the DLib software library [60]. The detector is very robust and accurate; it takes as input an RGB image and produces as output a bounding box of the area of the face. In the case of multiple detections, only the largest located face is used to run the reconstruction and the subsequent try-on process, thus avoiding unintentional runs of the try-on over faces that may be present in the background of the input picture.

Although face detection and reconstruction can work with images at a small resolution, in order to have sufficient information in the area of the eyes, the face is acquired at least with a resolution of 1000×1000 pixels.

For the 3D face reconstruction phase, based on the results in Section 4, we decided to use the PRNet [50] method. This method allows us to not enforce any particular restriction on the face pose or occlusions because the reconstruction process can work on almost every image depicting a human face. The input of the 3D face reconstruction module is the area of the face detected by the face detection module. The area is first resized to a 256×256 pixel RGB image and used as the input of the CNN. The output of the network is a vertex map, a 256×256 matrix where each element is a vertex of the reconstructed face. The face mesh is generated from the 65 K vertices in the regressed vertex map. Its texture image is generated by remapping the input image to the reconstructed mesh using the regressed vertices map. The resolution of the texture map is 512×512 pixels. This resolution is deemed of sufficient quality for our application.

If occlusions are present in the input image, the corresponding portions will be missing in the generated texture image. This is a limitation of the single image 3D face reconstruction approach. The face mesh is always complete, regardless of the initial face pose, but the face parts not visible to the camera are not textured correctly. This problem could be partially solved by using multiple face acquisitions from different points of view, and then using Structure From Motion approaches [31] so that the 3D face can then be reconstructed. However, this solution could annoy the user since it requires many images in order to have a faithful reconstruction. For this reason, we preferred to have a very fast and realistic 3D face reconstruction using a single image with acceptable coverage of the face texture. To ensure this, if the detected face has a very skewed pose, the user is asked to take another shot in a more frontal position.

To keep dimensions consistent between reconstructions, the vertex map is rotated according to the detected face pose to obtain the front view and is scaled in a cube of size 1×1×1 units in 3D world space, centered in the origin of a canonical right-handed global coordinate reference system. The 3D face model will be scaled to its final true size according to the parameters estimated by the fitting parameter estimation module, as described in Section 5.2.

In addition to the reconstructed 3D face, the 3D reconstruction module outputs other information needed to estimate the true size of the face: the 68 landmarks defined by the Multi-PIE landmark scheme [73] used for locating the eye regions, and the face key-points recovered from the vertex map, used for determining the fitting parameters for the eyeglass frame. This information is sent to the face size estimation module.

The average time required by the 3D reconstruction module is in the order of 0.62 s, of which 0.06 s is for face detection, 0.01 s is for mesh reconstruction, and the remaining time is for texture generation.

### 5.2. Face Size Estimation

Building a virtual try-on application requires that the wearable item(s) must be of the correct size when placed on the virtual model. With respect to the eyeglass application, this means that the glasses’ frame size must match the face size. Existing try-on applications often lack a proper face size estimation, and this cannot provide the user with a realistic try-on experience in terms of size fitting. To cope with this problem, some commercial applications estimate the face size using markers whose sizes are known, such as a credit card, placed on the forehead [21]. The use of markers is a common approach but it requires the user to inconveniently perform additional acquisitions and to have the proper marker at hand. Another problem with this approach is that the marker must be clearly visible, forcing the user to perform actions to deal with acquisition errors. This can negatively influence the overall experience, annoying the user.

To deal with the above-mentioned issues, we propose a markerless approach for estimating the face size. Our approach exploits facial features without requiring additional items to be acquired. Specifically, we use the diameter of the iris as a reference to determine the actual face size. By measuring the iris diameter in pixels, and knowing its average diameter in millimeters, we can estimate the actual face size. The iris diameter is related to the cornea diameter and, according to [74], the average white-to-white horizontal diameter is 11.71±0.42 mm.

**Figure 6:**
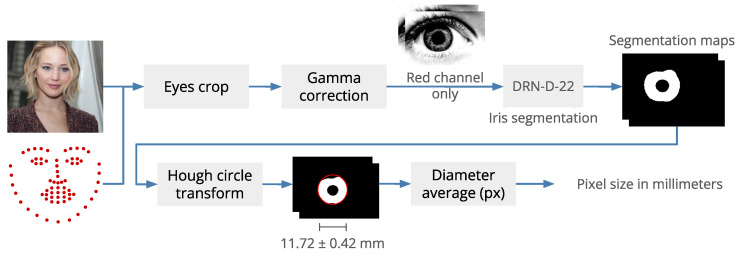
Face size estimation workflow.

The complete flow of our face size estimation process is summarized in Figure 6, and Algorithm 1 shows the algorithm. First, the eye location is identified using the landmarks provided by the 3D face reconstruction module. Then, we crop the eye regions, extract the red channel, and apply a gamma correction to enhance the visibility of the iris and pupil. Inspired by Kerrigan et al. [75], we then fed the processed eye crop into a DRN-D-22 network [76] to generate a segmentation map of the iris. Ideally, near-infrared images should be used, but this requires the adoption of additional hardware. Through our experimentation, we found that we can successfully use gray-level images as well. Finally, we apply the Hough Circle Transform to the segmentation map to fit a circle on the iris and find its radius in pixels. The size in millimeters of each pixel is finally computed as 11.71/2r, where *r* is the estimated iris radius in pixels.

This procedure is applied to both eyes, and the final iris size estimation is the average of the two values. If the calculation fails for one eye, the single value obtained is used. In the unfortunate event that the estimation fails for both eyes, the subsequent step of glasses fitting estimates the parameters to best fit the glasses on the user’s face without using the size information; in this case, the user is notified of such failure.

We can then align the 3D reconstruction on the input image and compute the face size using the distance between the ear key-points as the number of pixels between them multiplied by the size of each pixel in millimeters determined from the iris size. The whole face size estimation procedure requires 0.13 s on average.
**Algorithm 1:**Face size estimation algorithm.**Input:** the face image, the key-points detected on the face, and the estimated distance for the ears in pixels.**Output:** the estimated face size as the ear-to-ear distance in millimeters.1:**function**FaceSizeEstimation(input_img,kpts,ear2ear_pixels)2:    eye_kpts← fetch_eyes_kpts (kpts)               ▹ left, right eyes3:    padding←104:    px2mm←05:    detection_count←06:    **for** eye_kpts **in** eyes_kpts **do**7:        eye_img← crop_image input_image,eye_kpts,padding)8:        eye_img← gamma_correction eye_img)9:        eye_img←eye_img[:,:,0]          ▹ use red channel only10:        mask← drn_d_22_process eye_img)            ▹ predicts the iris segmentation mask11:        iris← detect_hough_circle mask)▹ fits a circle over the segmentation mask12:        mm←11.71/(2∗iris.radius)     ▹ size of a pixel in millimeters13:        **if** mm **is not** ’nan’ **then**              ▹ estimation successful14:           px2mm←px2mm+mm15:           detection_count←detection_count+116:    **if** detection_count>0 **then**17:        px2mm←px2mm/detection_count            ▹ average size of a pixel in millimeters18:    **else**19:        **raise** Exception(’Face size estimation failed!’)20:    **return** ear2ear_pixels∗px2mm          ▹ ear to ear distance in millimeters

### 5.3. Fitting Parameter Estimation

Once we have estimated the face size, we need to define the geometric transformation required to correctly place the glasses on the face. This is done by a fitting procedure that generates the transformation parameters required for rendering the face with the selected glasses frame. Since the glasses frames have different geometries, we need to perform the fitting for each selected one with respect to the current face geometry.

**Figure 7:**
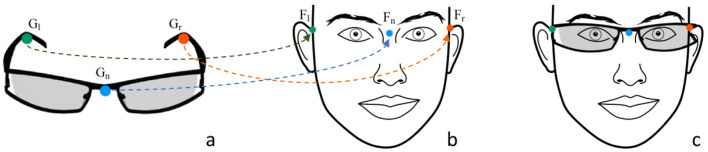
Example of key-points for glasses fitting. Nose key-points in blue, left ear key-points in green, right ear key-points in red. (**a**) Eyeglasses key-points sample. (**b**) Example of facial key-points. (**c**) Eyeglasses fitting result.

The parameters are estimated by finding the transformation that aligns the glasses frame on the reconstructed face when viewed from a frontal pose. The alignment is performed by using some of the facial landmarks extracted from the reconstructed 3D face (facial key-points) and key-points extracted from the glasses model (eyeglasses key-points). For this reason, we assume that all the available glasses models in the database are already annotated with these key-points, along with all the other relevant information. Specifically, we assume that the brand name, model name, preview image, width, and key-points are available. The required eyeglasses key-points are shown in Figure 7a. They correspond to the bridge location ($G_{n}$) and both the temples’ far end-points, where the glasses lean on the ears ($G_{l}$ and $G_{r}$). The corresponding facial key-points are shown in Figure 7b. These are extracted from the reconstructed 3D face. At the end of the fitting procedure, the two sets of key-points geometrically overlap and the glasses are fitted to the face, as shown in Figure 7c.

The fitting process requires several stages. First, it is necessary to determine the glasses’ position. This is done by using Equation (1), which computes the translation transformation that aligns the glasses bridge key-point $G_{n}$ with the face nose key-point $F_{n}$, which is used as a reference point:

(1) $$t=F_{n}-G_{n}$$

**Figure 8:**
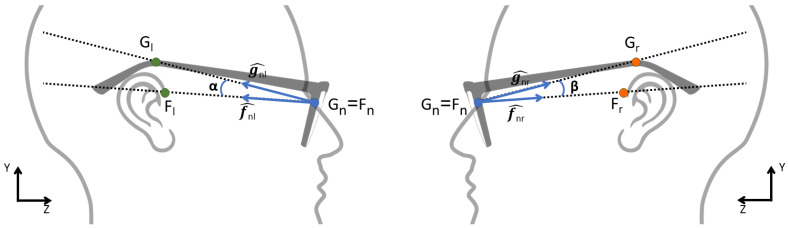
Eyeglasses pitch angle between ear and temple key-points.

Then, we need to correctly lay the glasses’ temples on the ears. This is done by computing the projection of the key-points on the 2D plane defined by the Z and Y axes, as shown in Figure 8 and Equation (2):

(2) $$\begin{array}{cc} \hat{g_{nl}} & =\frac{G_{l}-G_{n}}{|G_{l}-G_{n}|}\\ \hat{f_{nl}} & =\frac{F_{l}-F_{n}}{|F_{l}-F_{n}|}\\ \alpha & =\arctan2(|\hat{g_{nl}}\times \hat{f_{nl}}|,\;\hat{g_{nl}}\cdot \hat{f_{nl}})=\arctan2\left(\sin\alpha ,\cos\alpha \right) \end{array}$$

We compute the direction from the nose key-point ($F_{n}$) to the glasses left key-point ($G_{l}$). We normalize it to a unit vector, obtaining the unit directional vector that we indicate as $\hat{g_{nl}}$. Similarly, we define the unit vector $\hat{f_{nl}}$ that represents the direction from the nose key-point ($F_{n}$) to the left ear key-point ($F_{l}$). We then compute the angle α between the two unit vectors using the 2-argument arctangent, where the first argument is the norm of the cross product between $\hat{g_{nl}}$ and $\hat{f_{nl}}$, and the second argument is the dot product between them. With $\hat{g_{nl}}$ and $\hat{f_{nl}}$ being unitary vectors, the first argument corresponds to the sine of the angle α between them, and the second one to the cosine of α. In a similar way, a second angle β is computed using the direction from the nose key-point to the glasses right key-point ($\hat{g_{nr}}$), and the direction from the nose key-point to the right ear key-point ($\hat{f_{nr}}$). Finally, we build the rotation transformation around the X-axis needed to lean the glasses on the ears using the mean rotation angle between α and β.

To scale the glasses frame to match the face size, we take into account the face size estimation described in Section 5.2, and the difference between the mesh and face coordinate systems. In case of problems in the face size estimation, the scale is determined as the mean X-axis scale factor that best fits the temples on the ears.

**Figure 9:**
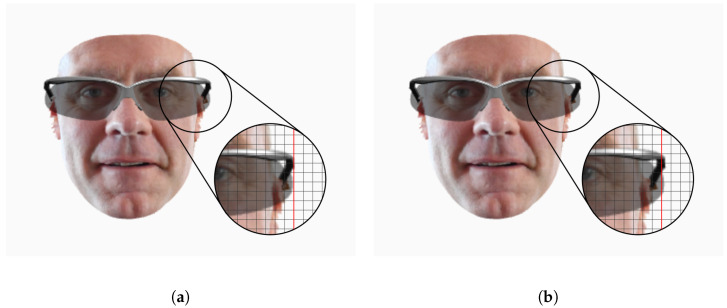
Examples of eyeglasses fitting using key-points and face size. (**a**) The glasses are scaled to match the key-points of the face. (**b**) The glasses are scaled according to the estimated face size and the real glasses sizes.

Figure 9 shows the difference between our fitting solution and a simple approach based on key-point correspondence. The key-point correspondence approach does not take into account face and eyeglasses dimensions. The glasses are simply scaled to make the key-points match the face ones (Figure 9a). With our approach instead, the glasses are slightly thicker on the face with respect to the key-point fitting approach (Figure 9b).

**Figure 10:**
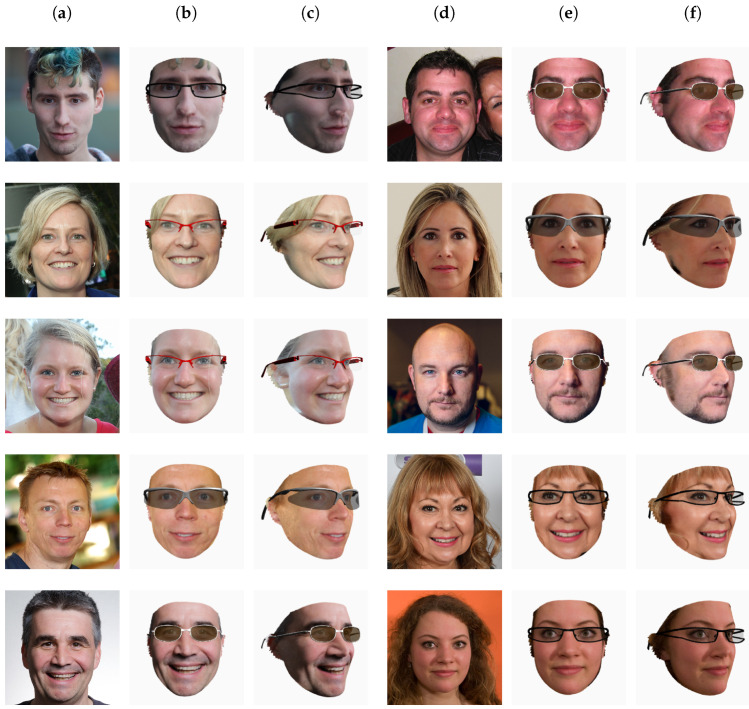
Examples of virtual try-on results on images from the FFHQ dataset [69]. Glasses models from [77]. (**a**) Input; (**b**) Front view; (**c**) Side view; (**d**) Input; (**e**) Front view; (**f**) Side view.

The overall fitting parameter estimation requires around 0.4 milliseconds for each eyeglasses model in the library. The 3D face mesh, the eyeglass model, and the fitting parameters are passed to the scene mixer module in order to render the final virtual 3D scene. Some virtual try-on results with different face pictures and eyeglasses models are visible in Figure 10, where the input images are taken from the FFHQ dataset [69], which provides high-quality images of human faces.

### 5.4. User Interface

We designed the virtual try-on user interface as a web application. The web application is responsible for image acquisition and user interaction. It also interacts with the back-end modules, specifically the face reconstruction and fitting parameter estimation modules, accessed as web services. The use of a web-based front-end allows us to deploy the try-on application on different devices with minimum system requirements, provided that modern web browsers are available.

The user uploads a picture or snapshot of his face using the web browser, along with the information about the glasses frames desired. The image is received by the server, and processed by the designed processing pipeline. The result is returned to the browser, allowing the user to see the glasses from different points of view.

The web application has been implemented using a set of open-source tools and frameworks. Specifically, we used *Vue.js* [78] as our core JavaScript framework, *Dropzone.js* [79] as the image upload handler, and *babylon.js* [80] as an advanced 3D rendering framework to manage Physically-Based Rendering (PBR) Materials and lens transparency.

The 3D models of the face, as well as those of the glasses, are exchanged using the Wavefront OBJ file format accompanied by the material definition file MTL and the texture images. While the 3D face model is automatically loaded shortly after the try-on back-end completes the processing, the 3D glasses models are dynamically loaded based on the user’s selection. Since the web application must still be usable on low-end devices and slow internet connections, the 3D models of the glasses have been optimized to reduce the size of the files to be transferred, in order to keep loading times below one second in most cases. For the glasses models, in addition to the geometry and the materials, the application also loads a JSON file containing the translation, rotation, and scale parameters to render them correctly on the 3D face.

**Figure 11:**
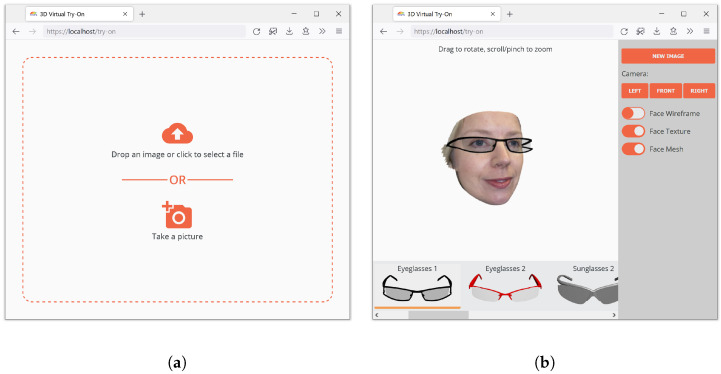
Screen captures of the virtual try-on web application user interface. (**a**) Image upload interface; (**b**) virtual try-on interface.

Figure 11 shows the user interface, which is composed of two main pages; the first is for face image upload or acquisition, and the second one is to display the 3D virtual try-on. The displayed acquisition interface is for desktop/mobile devices. If the application is installed in physical stores, a continuous video acquisition can be implemented, as described in the previous sections.

The majority of the screen area is used to render the 3D face and glasses. The list of glasses available for try-on is displayed on the bottom of the screen, and a few controls for the rendering and some predefined camera positions are available in the right sidebar. If the user’s device has a screen with limited size (e.g., a smartphone), the sidebar starts in a collapsed state to leave sufficient space for the try-on rendering. The face can be rotated by dragging, and the zoom level can be adjusted by using pinching or scrolling, depending on whether the device supports touch controls or not.

## 6. Try-On User Experience

To evaluate the efficacy of the application, we performed a standard usability test [81] with a panel of users. A total of 16 subjects of different age, expertise, and educational background were selected.

The experiment was conducted using a 10-inch tablet where the user interacts with a touch interface and a desktop PC where the user interacts with the mouse. The participants were randomly split between these two interaction modalities. Before starting the usability test, we briefly described the application scope to the users. They were then observed using the application to perform a virtual try-on session by taking a picture of themselves. No time limit was imposed. At the end of the test session, we asked each user to judge his experience by filling in a questionnaire. The questionnaire was the standard System Usability Scale (SUS) questionnaire developed by John Brooke [82]. In order to gain more insights into the application, we also asked the user to rate (from 1 to 5): the precision of glasses fitting, the realism of the 3D view, the usefulness of such a 3D view, and their interest in using this application if made publicly available. Finally, we also collected some free comments from the users.

Results of the SUS questionnaire are summarized in Table 5. As can be seen, the users rated the system very positively. The try-on was considered easy and very simple to use. No previous knowledge or experience was necessary to approach it. By applying the standard procedure [82], our application obtained an overall SUS score of approximately 90 out of 100, which is considered above average. The average score is set to 68 from a study on 500 systems, as described in [83]. Table 6 shows the scores of the four additional questions specific to our try-on application. The virtual try-on application with the 3D visualization of the face and glasses received very high appreciation, with scores of 4.9 out of 5. The users found this type of application enjoyable and useful, and were more than happy to try more. The technology was found to be engaging, although the overall visual quality of the rendering should be improved. This is demonstrated by the slightly lower score for the fitting precision and the realism, with a score of 3.8 and 3.7, respectively.

In general, the opinions of the users were quite positive. The majority of them appreciated the ease of use of the application. The users also commented on the usefulness of the 3D visualization and lamented the lack of this visualization in other online try-on systems. Finally, the automatic fitting of the glasses frame was appreciated. However, some users suggested that we allow a manual resize and position of the frame. This could be interesting for a vendor that personalizes glasses frames or even produces custom eyeglasses to meet the user’s preferences. Many users requested the possibility to integrate the application with social media in order to share their selection.

Users also pointed out some problems about the application. The main concern was the lack of hair and neck in the face model. These are limitations of the actual 3D reconstruction model used that are common to many other reconstruction approaches. One way to cope with the hair problem is to use more recent 3D reconstruction approaches, such as the one proposed by Xu et al. [84], which tries to also reconstruct the hair. However, these approaches are quite limited, and the hair is not fully reconstructed. Missing chunks of hair may annoy the user more than having no hair. Another way to cope with the hair is to employ a dedicated CNN [85]. This could potentially solve the problem at the cost of adding computational complexity and time. Another common opinion is that the 3D face mesh needs improvements in both geometry and texture. As before, we can use more recent 3D reconstruction approaches and super-resolution techniques to cope with these issues. Finally, some users suggested that adding highlights and reflections on the lenses would make the 3D model more realistic and appealing. This can be obtained by adjusting the properties of the materials and incorporating shaders in the rendering engine used to display the models.

## 7. Conclusions

In this paper, we presented a web application for an eyewear virtual try-on. After a critical analysis of existing eyewear virtual try-on applications that aimed to identify their limitations, we introduced the workflow of a generic virtual try-on system and described the main modules used to provide the try-on experience.

Since 3D face reconstruction is a key component of our try-on application, we conducted an extensive analysis of the state-of-the-art methods to evaluate their design, complexity, geometry reconstruction errors, and reconstructed texture quality. After such an evaluation, we selected PRNet as the method for 3D face reconstruction in our virtual try-on.

Our application uses a customized version of this blueprint to overcome the identified limitations of existing virtual try-on applications for face accessories: first, the eyeglasses virtual try-on is performed in the 3D space using a 3D model of the user’s face, reconstructed from a single input picture, allowing the user to interact with the application in a deferred mode and giving the possibility of observing the results from different points of view. Second, the glasses fitting takes into account the actual size of the glasses frames and the actual size of the face of the user, providing a realistic size fitting. The user’s face size is estimated with a markerless approach that exploits the iris diameter, thus removing additional acquisition steps as required by other applications.

We also performed a usability study of our eyewear virtual try-on application. The results show that the application is engaging and easy to use; the users appreciated the full 3D try-on and expressed willingness to use the proposed application if made publicly available. Future works can overcome some limitations observed in the usability study, such as the global quality of the rendering, which should be improved by providing better and complete 3D face reconstruction, and enhanced textures. Another possible extension is that of designing a processing pipeline capable of handling the possible presence of occlusions and self-occlusions in the input picture: while the face geometry reconstruction is possible even in the case of occlusions or self-occlusions, the texture will present missing parts. Restoration techniques such as [86,87] can be used to recover the missing portions, or the application may ask the user to provide a non-occluded face picture. Finally, the application can take advantage of better integration with online stores and social media services. Providing the try-on experience through a mobile application will also improve the integration with user devices: in this case, the new front-end mobile application can use the existing back-end web service.

## Figures and Tables

**Table 1:** Comparison of the main features of eyeglasses virtual try-on applications.

Applications	Input	Output	3D Glasses	Size Fitting	Markerless
Ditto [21]	video	images	✔	✔	—
Glassify [22]	image	image	—	—	✔
YouCam [23]	image	image	—	—	✔
Jeeliz [25]	video/image	video/image	✔	—	✔
Memory Mirror [24]	video	video	—	—	—
Virtual Mirror [26]	video	video	✔	—	✓
VirtualEyes [27]	image	image	✔	—	—
**Ours**	image	3D	✔	✔	✔

**Table 2:** Main characteristics of 3D face reconstruction methods in the state-of-the-art. SR: Shape Regression, MM: Morphable Model, S: single image, M: multiple images, G: geometry, T: texture, BM: BumpMap, BFM: Basel Face Model, DRN: Dilated Residual Network. TF: TensorFlow, PT: PyTorch.

Method	Year	Category	Input	Face	Network	Output	F.work
				Detection	Backbone		
PRNet [50]	2018	SR	S	DLib	U-Net	G + T	TF
Extreme3D [37]	2018	MM/BFM	S	DLib	ResNet	G + BM	PT
Deep3DFace [41]	2019	MM/BFM	S/M	External *	ResNet	G + T	TF
RingNet [42]	2019	MM/FLAME	S	External ^†^	ResNet	G + T	TF
DECA [46]	2021	MM/FLAME	S	FaceNet ^o^	ResNet	G + T	PT
3DDFAV2 [45]	2021	MM/BFM	S	FaceBoxes ^x^	MobileNetv3	G + T	PT
3DSFMFace [53]	2021	SR	S/M	External ^‡^	ResNet	G + T ^§^	PT
INORig [47]	2021	MM/BFM	M	S3FD ^+^	DRN	G + T	PT

* The method requires 5 facial landmarks along with the source image; † The method uses loosely cropped face image as input; o The method uses a fast version of MTCNN [66] for face detection; x The method uses FaceBoxes face detector [67]; ‡ The method requires that the face is segmented from the background; § The method outputs a colored point cloud; + The method uses the Single Shot Scale-Invariant Face Detector [68].

**Table 3:** Evaluation results of 3D face reconstruction methods in the state-of-the-art. All the timings are computed on 101 images, discarding the execution times of the first one in order to simulate a hot start condition for the system. Hardware used: Intel Core i7 7700 CPU and NVIDIA Quadro RTX 6000 GPU.

Method	Time Min	Time Max	Time Median	Time Mean	Time Std	Memory	GPU Memory
	(Seconds)	(Seconds)	(Seconds)	(Seconds)	(Seconds)	(MB)	(MB)
PRNet [50]	0.736	0.808	0.751	0.749	0.010	2361	1161
Extreme3D * [37]	15.564	15.840	15.604	15.598	0.031	1968	1925
Deep3DFace ^†^ [41]	0.582	0.627	0.592	0.591	0.009	2867	1235
RingNet [42]	0.741	0.826	0.789	0.789	0.016	3108	23,035 ^o^
DECA [46]	0.850	1.361	0.893	0.883	0.055	3022	18,227 ^+^
3DDFAV2 ^x^ [45]	0.740	0.798	0.770	0.769	0.009	3290	4683
3DSFMFace ^‡^ [53]	0.349	0.636	0.438	0.435	0.053	3260	1379
INORig ^§^ [47]	2.901	3.228	3.049	3.037	0.081	4253	21,691 ^+^

* CuDNN disabled due to incompatibilities with the GPU; † Added face detector and 5-point descriptor from DLIB as a preprocessing step; o The method seems to allocate all the available memory on the GPU even if the behavior is explicitly disabled; x The method has been modified to reconstruct only one face even if more are detected in the input image, “–onnx” flag not used; ‡ Face segmentation done manually and not included in the run-times; § For each image, the reconstruction is performed on the pair input image and its horizontal flip; + The peak memory usage is a short spike.

**Table 4:** Geometry evaluation results of 3D face reconstruction methods in the state-of-the-art.

Method	Median	Mean	Std	NoW Median ^†^	NoW Mean ^†^	NoW Std ^†^
	(mm)	(mm)	(mm)	(mm)	(mm)	(mm)
PRNet [50]	1.50	1.58	0.45	1.50	1.98	1.88
Extreme3D [37]	1.83	1.93	0.24	-	-	-
Deep3DFace [41]	1.35	1.50	0.45	1.11	1.41	1.21
RingNet [42]	1.46	1.43	0.16	1.21	1.53	1.31
DECA * [46]	1.30	1.46	0.34	1.09	1.38	1.18
3DDFAV2 [45]	1.66	1.65	0.41	1.23	1.57	1.39
INORig [47]	1.51	1.51	0.24	-	1.33 ^o^	0.28 ^o^

† Values from NoW Challenge [72]; * Evaluation of the coarse 3D model; o Values from the INORig paper [47], not reported on the challenge website.

**Table 5:** System Usability Scale (SUS) results.

		Strongly				Strongly	Avg.	SUS
	Statement	Disagree				Agree		Score
		1	2	3	4	5		
1	I think that I would like to use this application frequently.	0	0	8	6	2	3.6	2.6
2	I found this application unnecessarily complex.	11	5	0	0	0	1.3	3.7
3	I thought this application was easy to use.	0	0	0	1	15	4.9	3.9
4	I think that I would need assistance to be able to use this application.	15	1	0	0	0	1.1	3.9
5	I found the various functions in this application were well integrated.	0	0	3	6	7	4.3	3.3
6	I thought there was too much inconsistency in this application.	15	1	0	0	0	1.1	3.9
7	I would imagine that most people would learn to use this application very quickly.	0	0	1	2	13	4.8	3.8
8	I found this application very cumbersome or awkward to use.	11	4	1	0	0	1.4	3.6
9	I felt very confident using this application.	0	0	0	8	8	4.5	3.5
10	I needed to learn a lot of things before I could get going with this application.	16	0	0	0	0	1.0	4.0

**Table 6:** Application-specific question results.

Feature	Average Rating (Range 1–5)
Glasses fitting precision	3.8
Realism of the 3D view	3.7
Usefulness of the 3D view	4.9
Favorable to virtual try-on	4.8
